# Transcription strategies related to photosynthesis and nitrogen metabolism of wheat in response to nitrogen deficiency

**DOI:** 10.1186/s12870-020-02662-3

**Published:** 2020-10-01

**Authors:** Xin Liu, Chengmiao Yin, Li Xiang, Weitao Jiang, Shaozhuo Xu, Zhiquan Mao

**Affiliations:** 1 State Key Laboratory of Crop Biology, College of Agronomy, Shandong Agricultural University, Taian, 271018 Shandong China; 2 ShanDong Shofine Seed Technology Co., Ltd., Jiangxiang, 272400 Shandong China; 3 State Key Laboratory of Crop Biology, College of Horticultural Science and Engineering, Shandong Agricultural University, Taian, 271018 Shandong China

**Keywords:** Nitrogen deficiency, Nitrogen metabolism, Photosynthesis, Transcriptome, Wheat

## Abstract

**Background:**

Agricultural yield is closely associated with nitrogen application. Thus, reducing the application of nitrogen without affecting agricultural production remains a challenging task. To understand the metabolic, physiological, and morphological response of wheat (*Triticum aestivum*) to nitrogen deficiency, it is crucial to identify the genes involved in the activated signaling pathways.

**Results:**

We conducted a hydroponic experiment using a complete nutrient solution (N1) and a nutrient solution without nitrogen (N0). Wheat plants under nitrogen-deficient conditions (NDC) showed decreased crop height, leaf area, root volume, photosynthetic rate, crop weight, and increased root length, root surface area, root/shoot ratio. It indicates that nitrogen deficiency altered the phenotype of wheat plants. Furthermore, we performed a comprehensive analysis of the phenotype, transcriptome, GO pathways, and KEGG pathways of DEGs identified in wheat grown under NDC. It showed up-regulation of Exp (24), and Nrt (9) gene family members, which increased the nitrogen absorption and down-regulation of Pet (3), Psb (8), Nar (3), and Nir (1) gene family members hampered photosynthesis and nitrogen metabolism.

**Conclusions:**

We identified 48 candidate genes that were involved in improved photosynthesis and nitrogen metabolism in wheat plants grown under NDC. These genes may serve as molecular markers for genetic breeding of crops.

## Background

Excessive nitrogen application and low nitrogen utilization efficiency in winter wheat crops are challenging tasks across the world [1]. The low nitrogen utilization efficiency in wheat is primarily due to the excessive application of nitrogen fertilizer [2]. Besides, it causes environmental pollution and hampers the sustainable development of agriculture. On the premise of ensuring crop yield, reduced nitrogen application demands an urgent investigation. An in-depth understanding of physiological, metabolic, and morphological processes in wheat using molecular breeding methods can improve crop yield and nitrogen use efficiency (NUE) [3, 4] in wheat plants grown under nitrogen-deficient conditions (NDC).

A detailed understanding of the plant’s physiology, metabolism, and root canopy structure is crucial for improving crop yield and resource utilization efficiencies under stress conditions, such as shading, drought, or nutrition deficiency [5–7]. As shown in a previous study, reduced nitrogen application modified the root morphology and improved root architecture, which in turn increased the nitrogen absorption capacity and NUE [8], but it reduced the photosynthesis and metabolic rate [9, 10]. Thus, to improve the adaptability of the wheat plant to nitrogen deficiency, it is crucial to discern the physiological and metabolic processes of the wheat plant at the transcriptomic level.

Nitrogen deficiency alters the gene expression in plants. Nitrogen deficiency in barley plants induced the upregulation of HvNiR1, HvGS2, HvGLU2, downregulation of HvASN1 in the shoot, and upregulation of HvGLU2 in the root. Thus, it improved the adaptability of barley plants to nitrogen-deficiency [11]. The up-regulated alternative oxidase (AOX) increased the utilization of excessive sugar and balanced the carbon level under NDC [12–14]. Similarly, the GmCZ-SOD1 gene was highly induced in the roots of the soybean plant grown under NDC [2]. 1799 differentially expressed genes (DEGs) were identified in maize crops grown under NDC [11]. Although multiple transcriptomic studies have been performed on the wheat crop, genes associated with wheat crop’s physiology and metabolism under NDC remain unknown, demanding an in-depth investigation [15].

This study therefore conducted experiment which aimed to: (i) explore the physiological, metabolic and morphological changes of wheat under nitrogen deficiency condition; (ii) screen the differentially expressed genes (DEGs) from wheat transcriptome under nitrogen deficiency; (iii) after comprehensive analysis of transcription, metabolic pathway and phenotype of important physiological and metabolic processes, we try to find out the potential genes which can be promote wheat growth under nitrogen deficiency.

## Result

### Morphological and physiological changes in wheat grown under the nitrogen-deficient condition

**Fig. 1:**
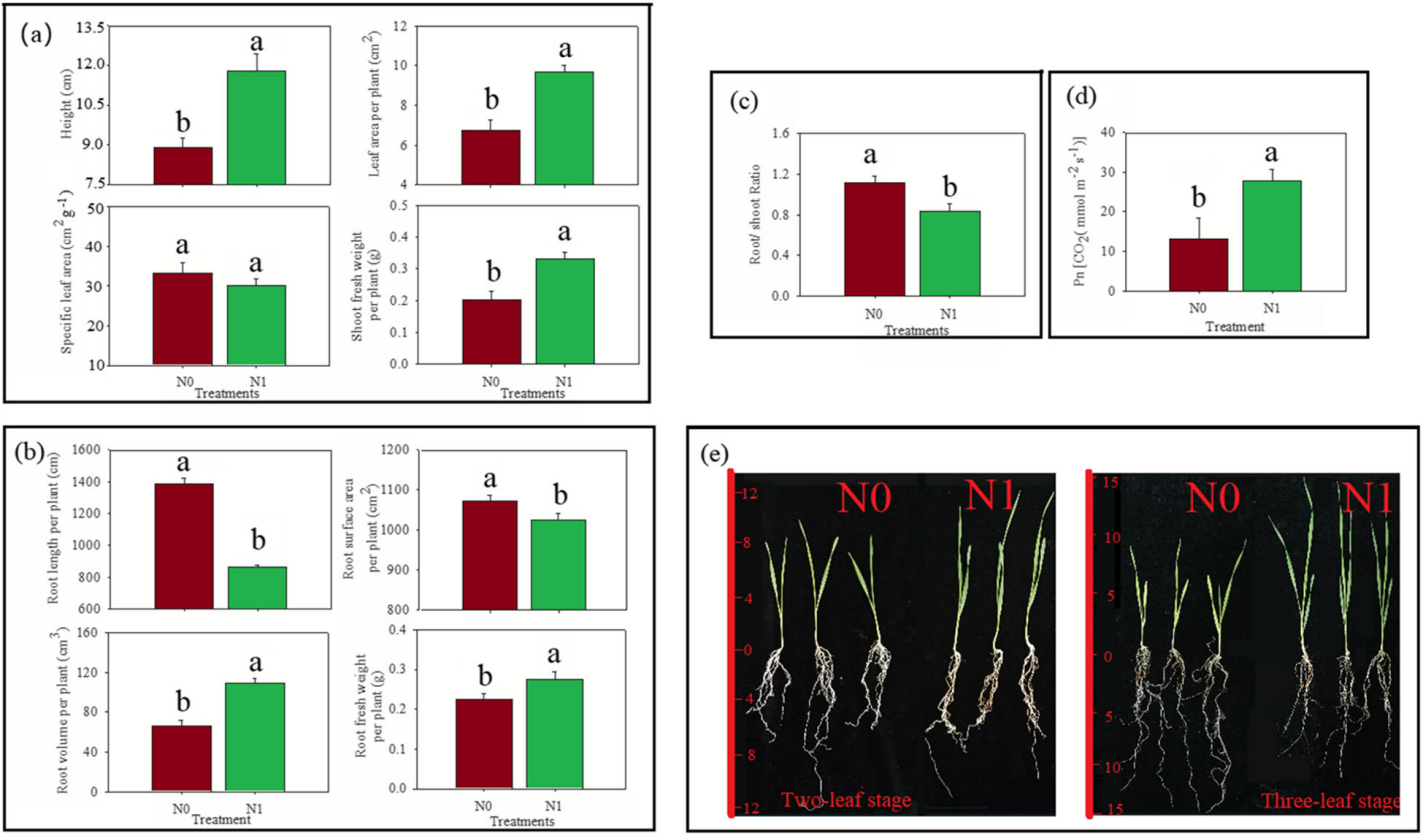
Effects of nitrogen content on winter wheat crop. **a** The shoot morphology, including crop height, leaf area per plant, specific leaf area, shoot fresh weight; **b** root morphology, including root length per plant, root surface area, root volume per plant, root fresh weight per plant; **c** root/shoot ratio; **d** net photosynthetic rate; **e** phenotypes, under normal nitrogen (N1) and nitrogen-deficient (N0) conditions. The root length per plant, root surface area, root volume per plant, root fresh weight per plant were the sum of all roots of one plant. Significance levels of differences between N0 and N1 group were estimated using the two-tailed t-test method. Different lowercase letters represent significant differences

The altered morphological and physiological states of wheat are depicted in Fig. 1. The height of the wheat plant in the N0 group was 0.75 times significantly lower than the wheat plants in the N1 group (Fig. 1a). The leaf area per plant of the wheat plants in the N0 group was 0.70 times significantly smaller than the wheat plants in the N1 group. However, no significant differences were observed in the specific leaf area of wheat plants in the N0 and N1 groups. The net photosynthetic rate (Pn) and fresh shoot weight of wheat plants in the N0 group were 0.47 and 0.61 times significantly lower, respectively, than the wheat plants in the N1 group (Fig. 1d). It showed that nitrogen deficiency led to reduced crop height, leaf area per plant, Pn, and fresh shoot weight in wheat plants.

The root length per plant of wheat plants in the N0 group was 1.61 times significantly more than wheat plants in the N1 group (Fig. 1b). Besides, the root volume per plant of wheat plants in the N0 group was 0.61 times lower than wheat plants in the N1 group. However, the root surface area per plant, fresh root weight, root shoot ratio of wheat plants in the N0 group was 1.04, 0.82, and 1.36 times higher, respectively, than wheat plants in the N1 group (Fig. 1c). It showed that nitrogen deficiency resulted in an increased root length, root surface area per plant, fresh root weight, root shoot ratio, and reduced root volume per plant.

### Global analysis of RNA-seq data of wheat plants grown under the nitrogen-deficient condition

The number of genes expressed in different parts of N0 group wheat plants was calculated to construct the stacked histogram (Fig. S1a). A total of 72,487–78,729 genes were identified in the wheat shoot of the N0 group, out of which 17,116–22,418 genes had FPKM (Fragments Per Kilobase of transcript per Million fragments mapped) values > 1. Besides, 63,273–64,413 genes were identified in the wheat root of the N0 group, out of which 27,785–29,233 genes had FPKM values > 1.

Principal component analysis (PCA) was applied to explore the relationship between samples by locating the samples at different dimensions (Fig. S1b). Less clustering distance indicated more identical samples. PCA1 reflected the difference in root and shoot, accounting for 99.41% of the total variation. Besides, shoot and root transcription differences between the N0 and N1 groups of wheat plants were deduced by PCA2 and PCA3, which accounted for 0.21 and 0.11% of the total variation. PCA3 reflected the root transcription difference between the N0 and N1 groups of wheat plants, accounting for a total variation of 0.11%.

**Fig. 2:**
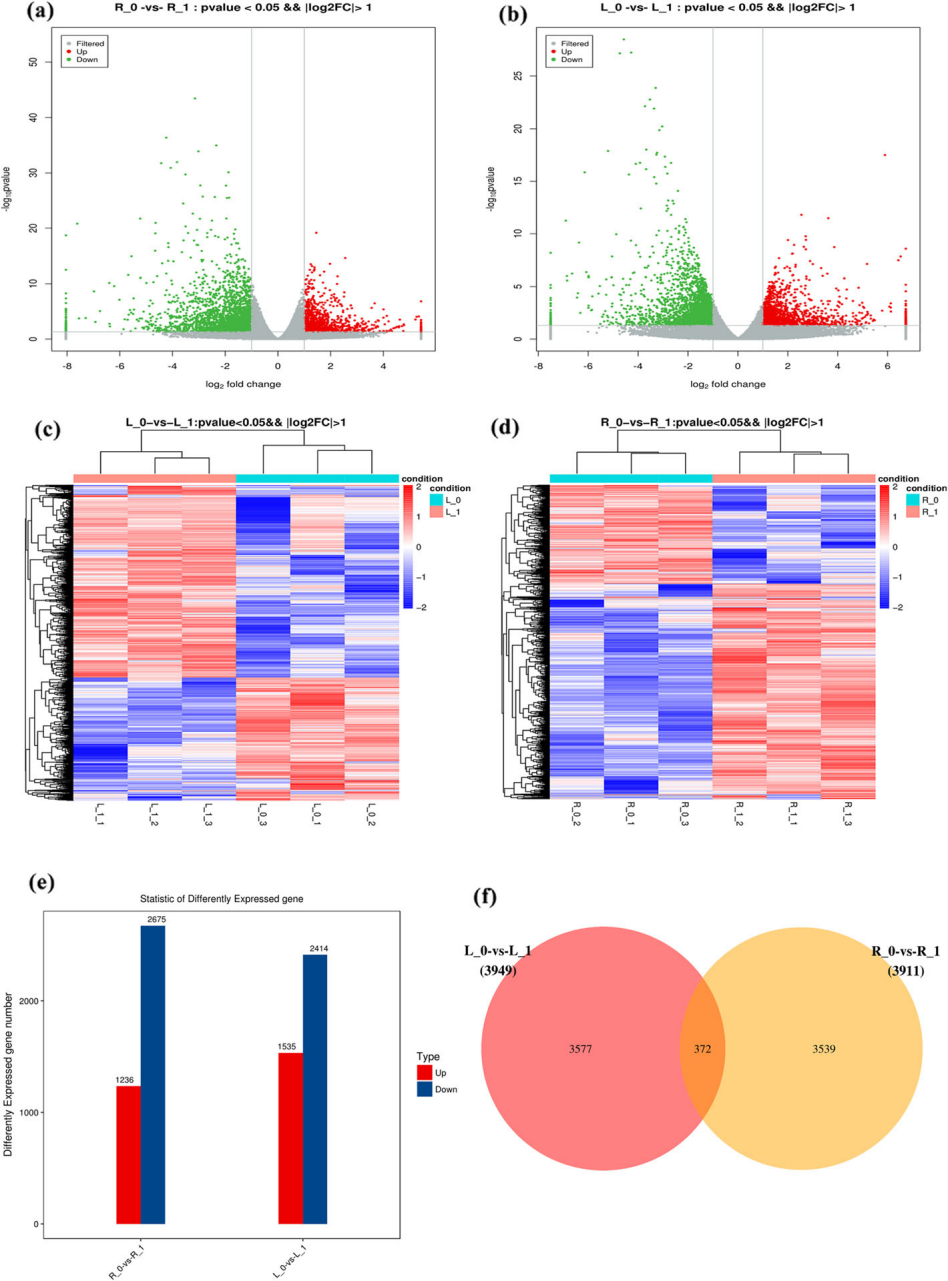
Volcanogram (**a** represents root, **b** represents shoot), cluster map (**c** indicates shoot, d indicates root), **e** number of differentially expressed genes in wheat, and **f** Venn map under nitrogen-deficient condition. R_0 and L_0 represent the root and shoot of N0 (nutrition solution without nitrogen) group of plants, respectively; R_1 and L_1 represent the root and shoot of N1 (complete nutrition solution) group of plants, respectively. In volcanogram (**a**, **b**), gray points were the genes with a non-significant difference, red and green points were the genes with significant differences; X-axis display of log_2_ foldchange (FC), and Y-axis display *p*-value. In the cluster map (**c**, **d**), red represent up-regulated and blue represent down-regulated protein-coding genes

The volcanogram (Fig. 2a, b) and cluster map (Fig. 2c, d) of *p*-values and log_2_FC were applied to screen the differentially expressed genes (DEGs) in the N0 group of wheat plants as compared to the control (N1) wheat plants. We identified a total of 3949 DEGs in the shoots of wheat plants grown under NDC, out of which 1535 were up-regulated, and 2414 were down-regulated. Besides, we identified a total of 3911 DEGs in roots of wheat plants grown under NDC, 1236 of which were up-regulated, and 2675 were down-regulated (Fig. 2e). The Venn map (Fig. 2f) revealed that 1535 DEGs were up-regulated and 2414 were down-regulated in both shoot and root of wheat plants grown under NDC, and a total of 372 DEGs were identified in roots and shoot.

### Functional analysis of DEGs identified in wheat grown under the nitrogen-deficient condition

1205 up-regulated genes and 1888 down-regulated genes in shoots, while 961 up-regulated genes and 1883 down-regulated genes identified in roots of wheat plants grown under NDC, were enriched in Gene Ontology (GO) analysis (Fig. S2). The enriched genes were classified into 3 major classes and 64 sub-classes, and some of these genes belonged to two or more categories. Cellular process, metabolic process, binding, and catalytic activity were the top enriched categories, which included more than 980 DEGs (Table 1).

**Table 1:** The number of differentially expressed genes (DEGs) in the four pathways with the largest number of genes under the nitrogen-deficient condition

	GO category	Sub-category	DEGs-Up	DEGs-Down
Shoot	Biological process	Cellular process	385	947
	Biological process	Metabolic process	498	1029
	Molecular function	Binding	672	1031
	Molecular function	Catalytic activity	560	854
Root	Biological process	Cellular process	312	669
	Biological process	Metabolic process	390	891
	Molecular function	Binding	527	893
	Molecular function	Catalytic activity	429	936

**Fig. 3:**
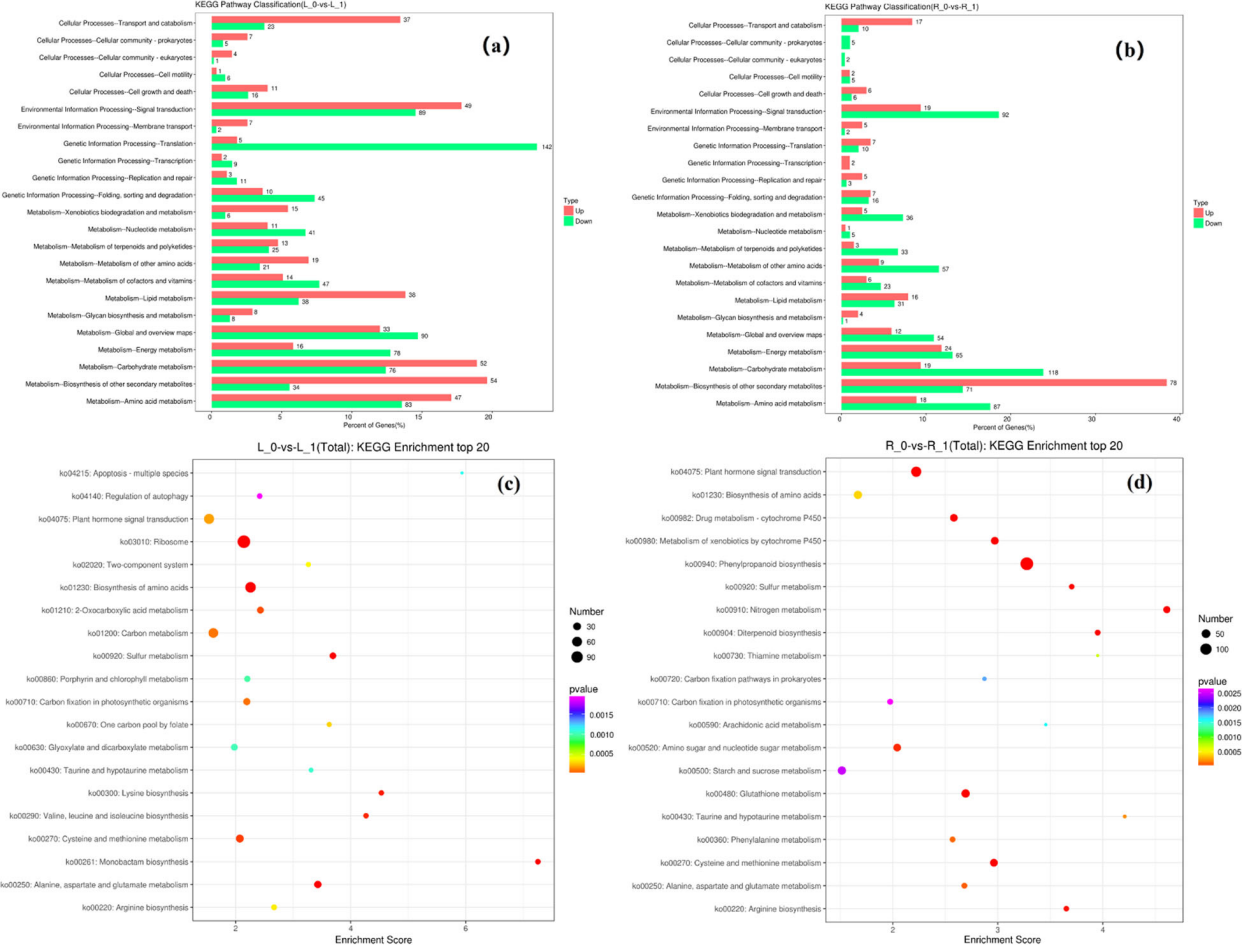
The KEGG classification of differentially expressed genes (DEGs) in (**a**) shoot and (**b**) root under nitrogen-deficient condition. The red column and green column represent up-regulated and down-regulated DEGs, respectively. The top 20 of KEGG pathways in (**c**) shoot and (**d**) root, under nitrogen-deficient condition. R_0 and L_0 represent the root and shoot of N0 (nutrition solution without nitrogen) group of plants, respectively; R_1 and L_1 represent the root and shoot of N1 (complete nutrition solution) group of plants, respectively

We performed KEGG pathway enrichment analysis of (Fig. 3a, b) DEGs from shoots and roots of wheat plants grown under NDC, and the pathways that showed enrichment of the highest number of DEGs are discussed here. Root DEGs showed enrichment of the gene information processing-translation pathway (142 down-regulated genes), and metabolism-biosynthesis of other secondary metabolites pathway (54 up-regulated genes). Root DEG’s KEGG pathway analysis led to the enrichment of the metabolism-carbohydrate metabolism pathway (118 down-regulated genes) and the metabolism-biosynthesis of other secondary metabolites pathway (78 up-regulated genes). Shoot DEG’s KEGG pathway analysis showed enrichment of monobactam biosynthesis (Fig. 3c) and the nitrogen metabolism pathway (Fig. 3d).

### Analysis of gene families associated with cellular process

**Fig. 4:**
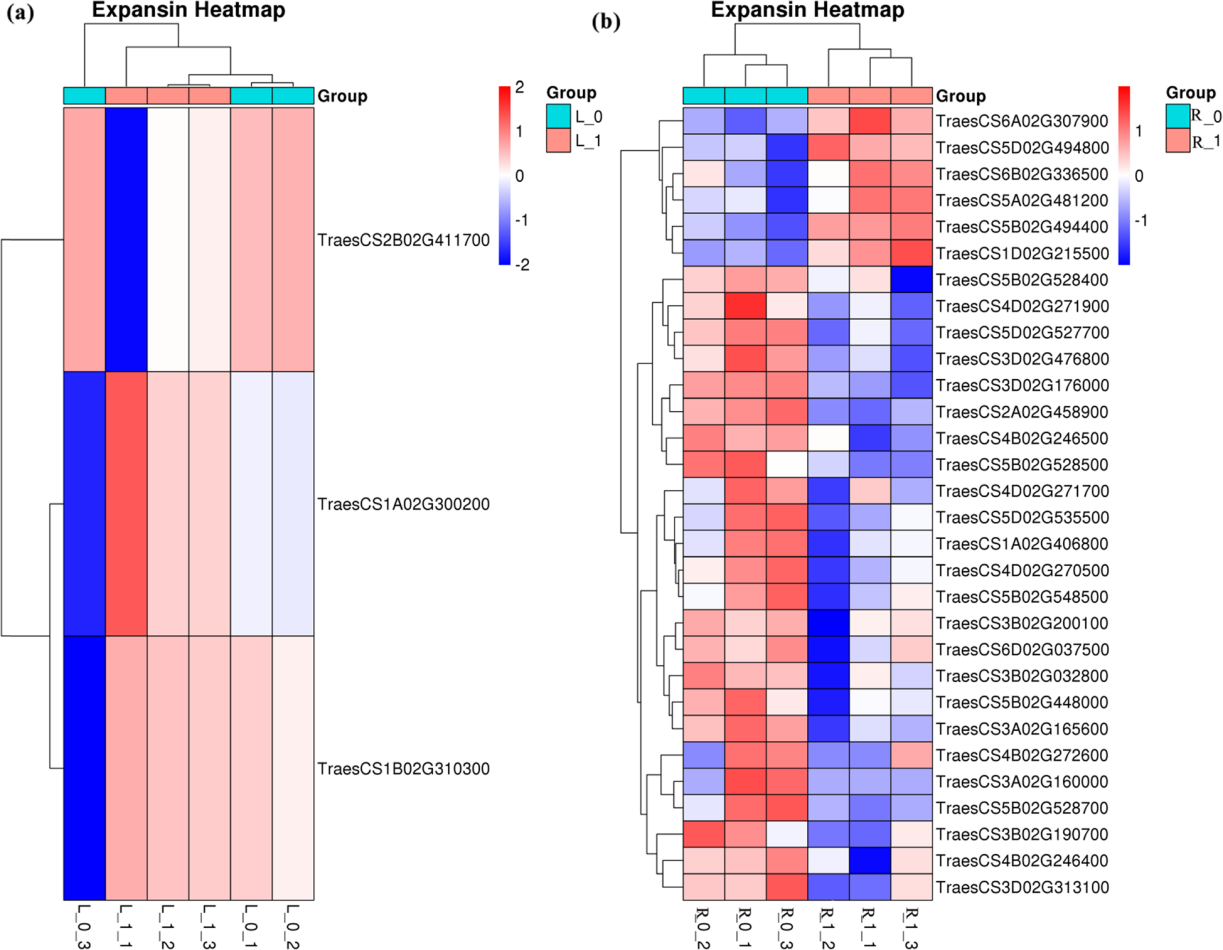
The heatmaps of differentially expressed genes (DEGs) of wheat (**a**) shoot and (**b**) root grown under nitrogen-deficient condition, member of expansin family. The DEGs were selected by *p*-value<0.05 and − 1<log_2_FC<1. R_0 and L_0 represent the root and shoot of N0 (nutrition solution without nitrogen) group of the wheat plant, respectively; R_1 and L_1 represent the root and shoot of N1 (complete nutrition solution) group of the wheat plant, respectively

Expansin family members primarily belong to the GO category-cellular process. 3 DEGs in shoot of wheat plant grown under NDC belonged to (Fig. 4) expansin family, including TreasCS2B02G411700 (up-regulated), TreasCS1A02G30020 (down-regulated) and TreasCS1B02G310300 (down-regulated). Also, 6 down-regulated genes (TreasCS6A02G307900 and so on) and 24 up-regulated genes (TreasCS5B02G528400 and so on) in roots of the wheat plant grown under NDC belonged to the expansin family.

### Analysis of gene families associated with metabolic process

**Fig. 5:**
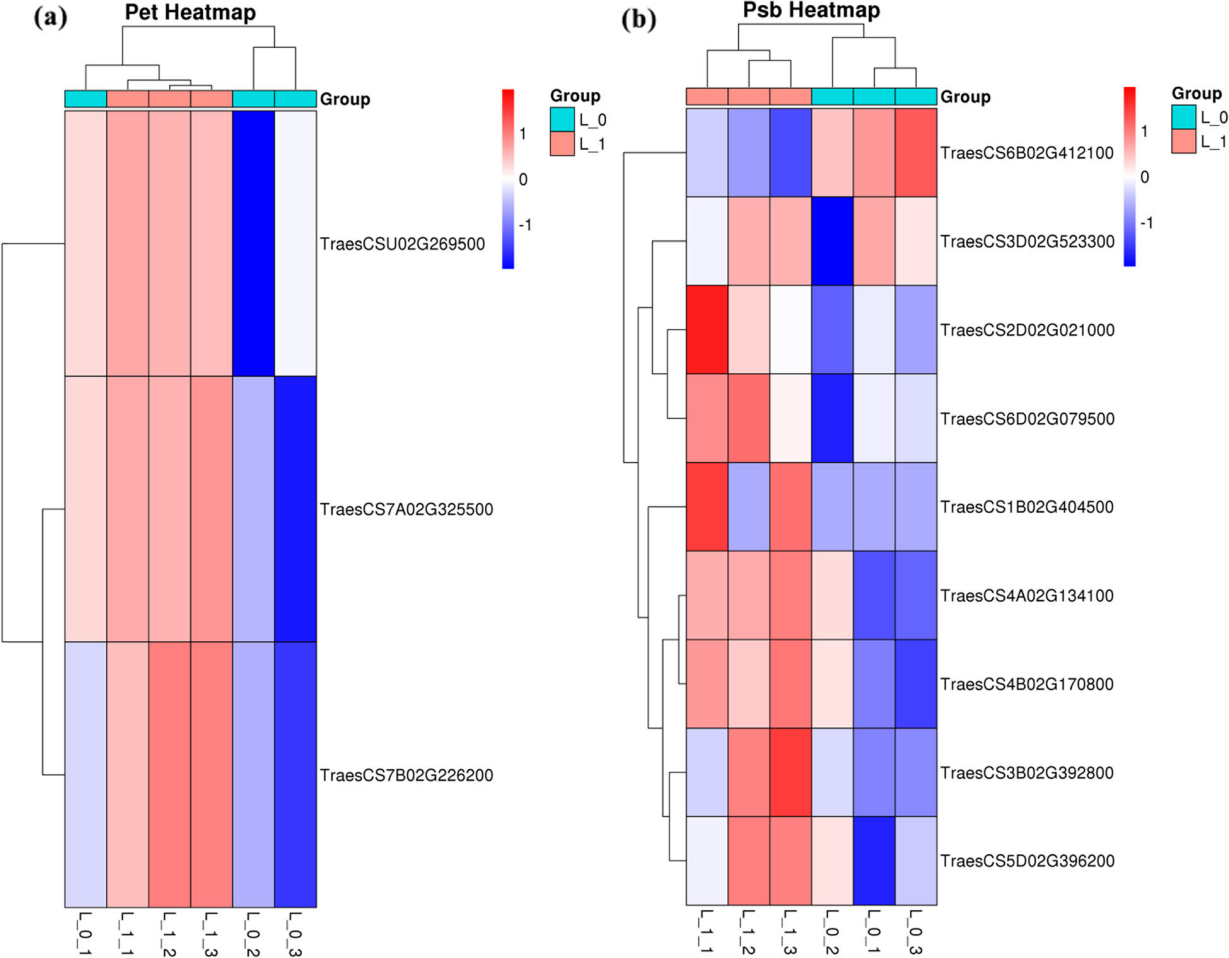
Heatmaps of differentially expressed genes (DEGs), member of the Pet and Psb family, from the shoot of the nitrogen-deficient wheat plant. A cut-off of p-value<0.05 and − 1<log_2_FC<1 was employed to screen DEGs. L_0 represent shoot of N0 (nutrition solution without nitrogen) group of wheat plants, L_1 represent shoot of N1 (complete nutrition solution) group of wheat plants

Pet and Psb family members serve as crucial photosystem members in the wheat shoot and belong to the GO category-metabolic process. In wheat plants grown under NDC, 3 down-regulated DEGs (Fig. 5) belonged to the Pet family (TreasCS7A02G325500 and so on), 8 down-regulated DEGs belonged to the Psb family (TreasCS3D02G523300 and so on), and 1 up-regulated DEG belonged to the Psb family (TreasCS6B02G412100).

**Fig. 6:**
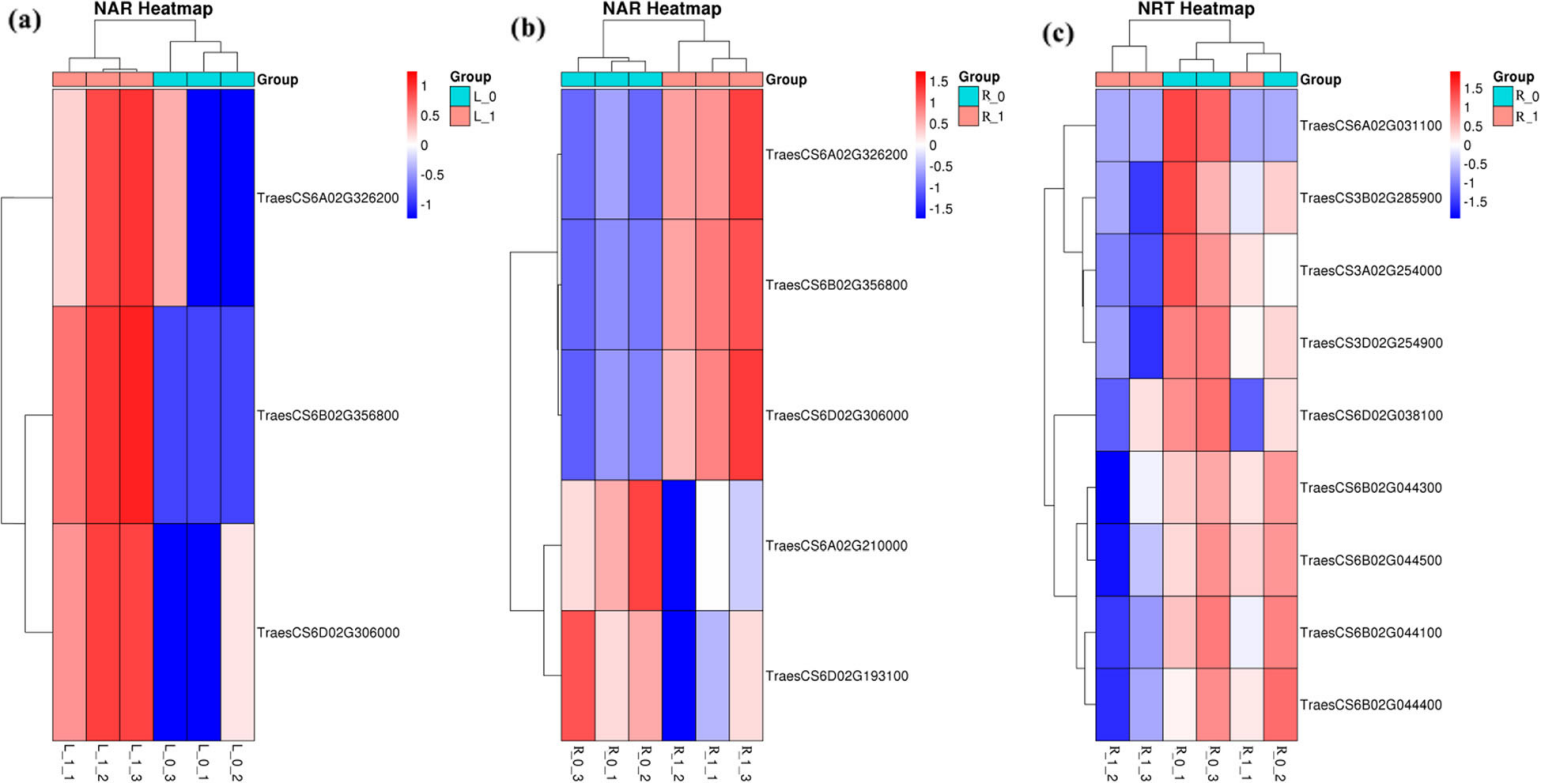
Heatmaps of differentially expressed genes (DEGs) in Nar (**a**) in the shoot, Nar in the root (**b**), and Nrt in the root (**c**) of nitrogen-deficient wheat plants. A cut-off of p-value<0.05 and − 1 <log_2_FC<1 was employed to screen DEGs. R_0 and L_0 represent the root and shoot of N0 (nutrition solution without nitrogen) group of wheat plants, respectively; R_1 and L_1 represent the root and shoot of the N1 (complete nutrition solution) group of wheat plants, respectively

Nar and Nrt family members are involved in nitrogen metabolism and belong to the GO category-metabolic process. In wheat plants grown under NDC, 3 down-regulated genes from root and shoot (Fig. 6) belonged to Nar family (TreasCS6A02G326200, TreasCS6B02G356800, and TreasCS6D02G306000), 2 up-regulated genes from root belonged to Nar family members (TreasCS6A02G210000 and TreasCS6D02G193100), and 9 up-regulated DEGs from root belonged to Nrt family (TreasCS6A02G031100).

### Validation of transcriptomic data

**Fig. 7:**
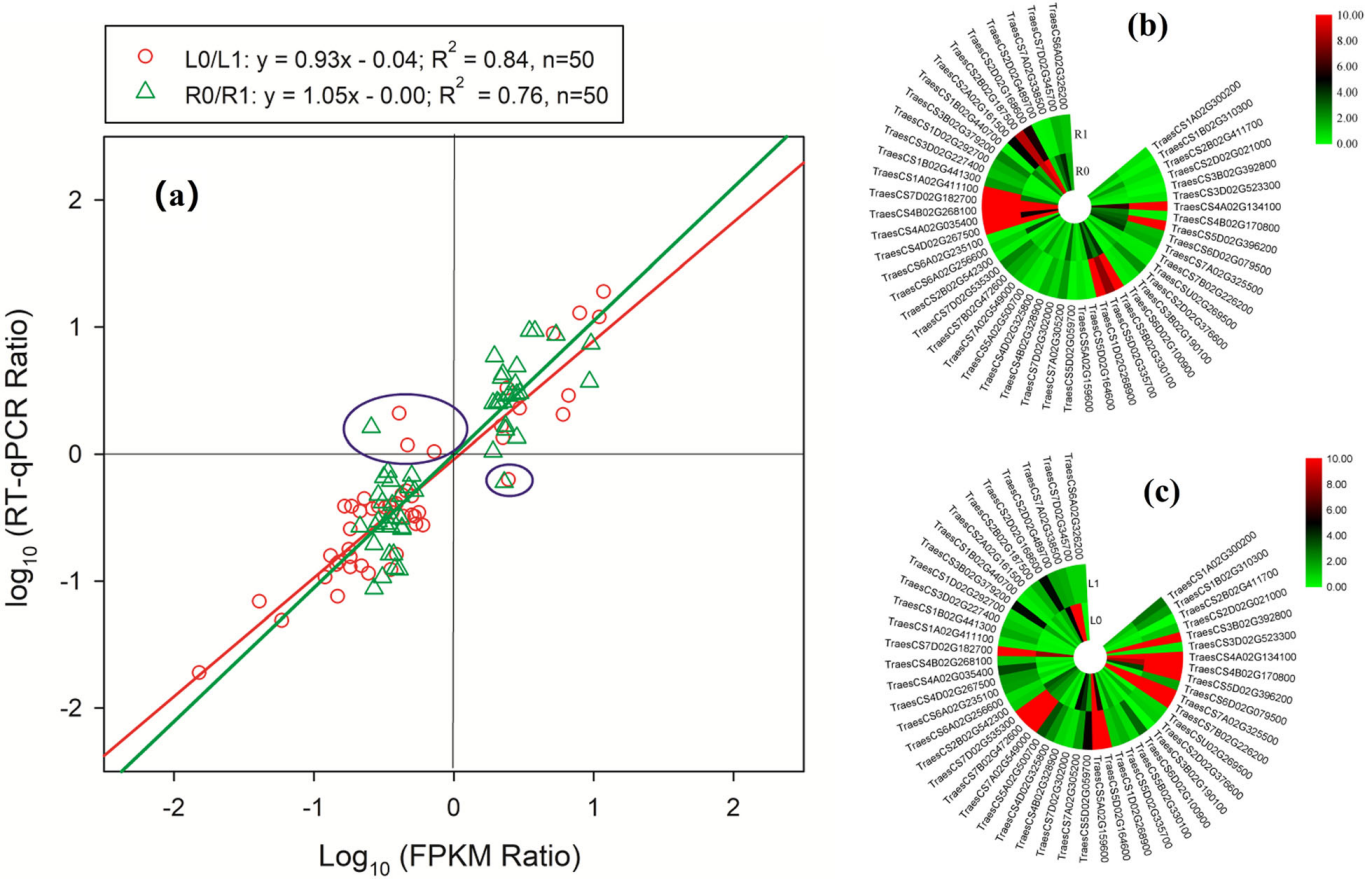
(**a**) The regression line of log_10_ (FPKM ratio) and log_10_ (RT-qPCR ratio) in shoot and root; the relative expression levels of 50 candidate genes in (**b**) root and (**c**) shoot, respectively. In Fig. 9a, the red circles represent the log_10_ (FPKM ratio) and log_10_ (RT-qPCR ratio) value of L0/L1, and the green triangles represent the log_10_ (FPKM ratio) and log_10_ (RT-qPCR ratio) value of R0/R1. The circles and triangles in the blue box represent the genes whose RT-qPCR results were inconsistent with the transcriptomic data. R0 and L0 represent the root and shoot of N0 (nutrition solution without nitrogen) group of nitrogen-deficient wheat plants, respectively; R1 and L1 represent the root and shoot of N1 (complete nutrition solution) group of wheat plants, respectively

As per the RT-qPCR based validation study, expression levels of 94 (46 shoot genes and 48 root genes) out of 100 candidate genes were in line with the FPKM values of transcriptomic data (Fig. 7a). It showed that around 94% of the transcriptomic data were reliable. The coefficients of X of regression lines were 0.93 and 1.05 for shoot and root, respectively, which indicated high accuracy of the transcriptomic data. The RT-qPCR data of 50 candidate genes from root and shoot are depicted in Fig. 7b, and Fig. 7c, respectively. The comparison between RT-qPCR and transcriptomic data of each gene can be queried using Table S1 and Table S2.

## Discussion

**Fig. 8:**
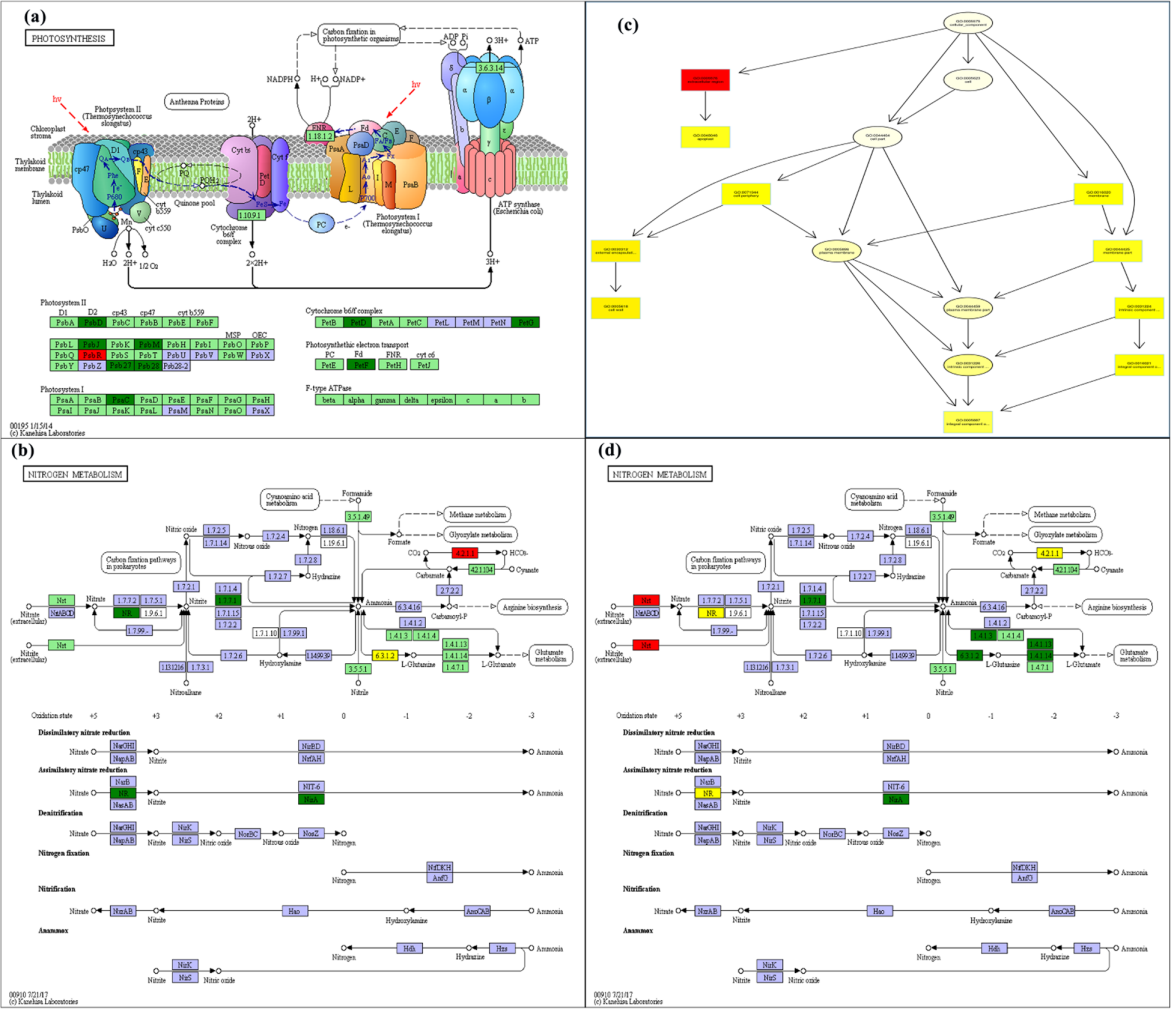
The KEGG pathway analysis led to the enrichment of DEGs from the wheat shoot in (**a**) photosynthesis pathway and (**b**) nitrogen metabolism pathway. The enrichment of DEGs in (b) the extracellular pathway as per the GO analysis, (**c**) the nitrogen metabolism pathway as per the KEGG analysis (**d**) identified in the root of the wheat plant. The red frame represents the up-regulated genes; the green frame represents the down-regulated genes

The altered morphology, metabolism, and physiology of wheat plants can be inferred from its transcriptomic data [16–18]. As per a previous report, in the photosynthesis pathway (Fig. 8a), the proteins coded by the Pet and Psb gene families were crucial components of cytochrome b6/f complex, photosynthetic electron transport, and photosystem II [19–23]. Previous studies showed that the inhibition of these proteins hampered the photosynthetic efficiency of plants [24, 25]. In the current study, genes belonging to Pet and Psb gene families were found to be downregulated. These down-regulated genes led to the inhibition of photosynthetic electron transport and the photosystem II pathway in wheat plants grown under NDC, which reduced the photosynthetic rate and energy metabolism.

Furthermore, DEGs identified in wheat plants grown under NDC were also enriched in the nitrogen metabolism pathway (Fig. 8b, d). DEGs belonging to Nar (nitrate reductase) gene family were involved in the nitrate-N reduction to nitrite-N process [26, 27]. DEGs belonging to Nir (Nitrite reductase) gene family were involved in the nitrite-N reduction to the ammonium-N process [28, 29]. Moreover, DEGs belonging to Nrt gene family were involved in the process of nitrogen transport from extracellular to intracellular process [30]. Moreover, as per the previous report, the Nrt family were found to be involved in root growth, flowering time, and transcriptional regulation of multiple physiological processes, hormonal and nitrate signaling [31–34]. The up-regulated DEGs, the member of Nir and Nrt gene families, increased the nutrient uptake in crops [35–37]. The expression levels of genes identified in the shoot of wheat grown under NDC, which belonged to Nar and Nir gene families, and the component of the nitrogen metabolism pathway was found to be down-regulated. Similarly, in the root, the expression levels of genes belonging to the Nir gene family were found to be down-regulated, which in turn mitigated the nitrogen metabolism pathway in both shoot and root. Interestingly, the expression of Nrt gene family members in nitrogen-deficient wheat root was up-regulated, which accelerated the movement of extracellular nitrogen into cells. The highest enrichment score of nitrogen metabolism pathway in wheat plant grown under NDC indicated that transcription differences had the highest influence on root nitrogen metabolism.

In the extracellular region pathway (Fig. 8c), the expansin gene family member increased the extensibility of the plant cell wall [38–40]. Previous studies stated that overexpressed expansin gene family members altered the crop morphology and improved the adaptability of crops to stress or low nutrition [41, 42]. For instance, the overexpressed TaEXPB23 altered the root system architecture of transgenic tobacco plants and improved the adaptability of plants to low phosphorus conditions [8]. In this study, under the NDC, the expression of expansin gene family members in root was up-regulated, which led to increased root length and surface area of wheat plants. The increased root length and surface area increased the nitrogen absorption efficiency of the wheat plants grown under NDC.

**Fig. 9:**
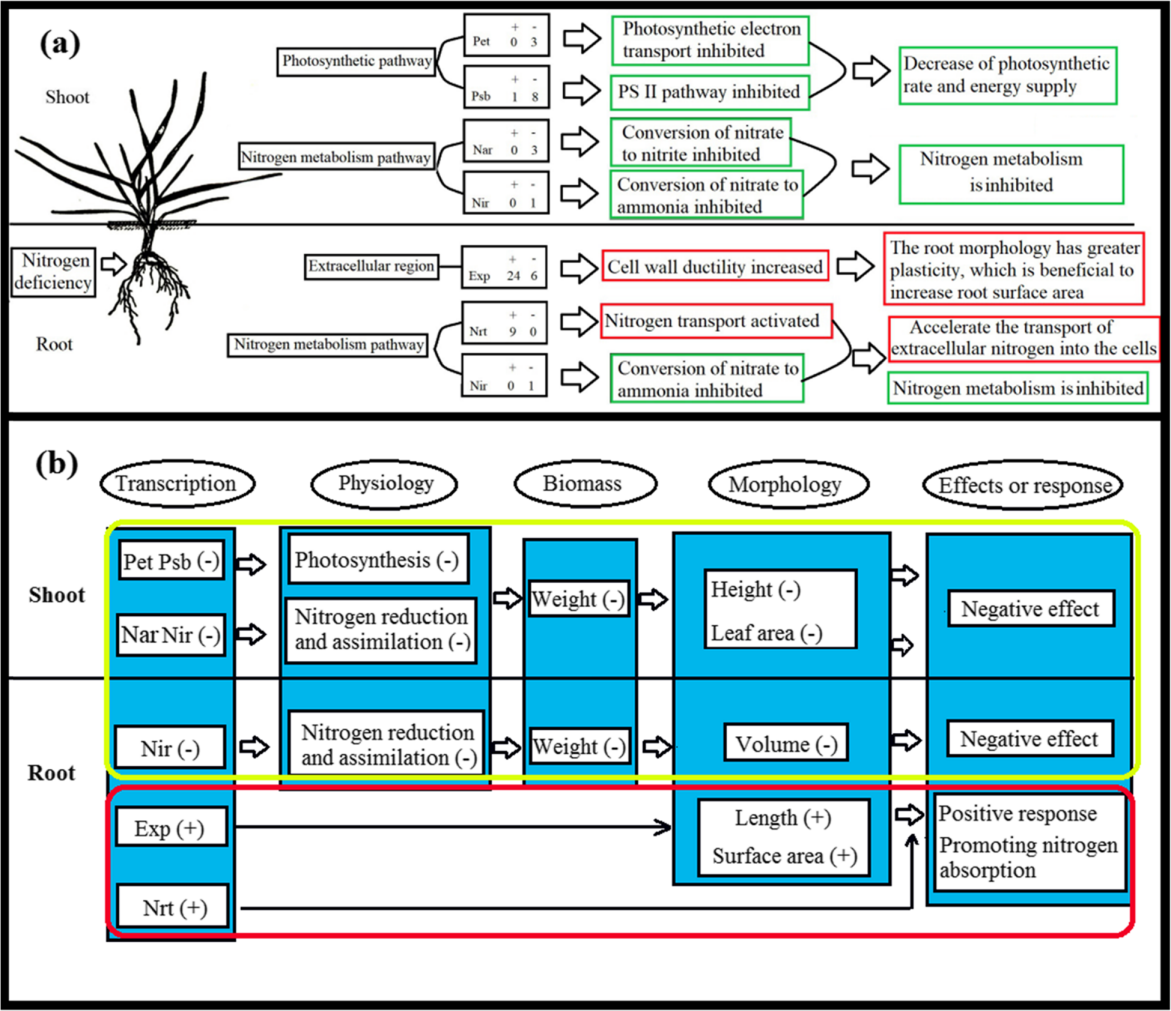
(**a**) The chart of pathways as part of plants response to nitrogen-deficient condition; (**b**) the chart depicts the correlation between gene transcription, physiology, biomass, morphology, and their effects or response under nitrogen deficiency. “+” indicates up-regulated or increased; “-” indicates down-regulated or decreased

The transcription level of plants changes in accordance with the external environmental conditions [39, 43], which in turn affect the protein levels and metabolism process, culminating in matter accumulation and morphological changes [8, 44]. Some responses improve the adaptability of crops to the external environment. Moreover, the differential expression of genes in roots and leaves leads to different effects. The up-regulated expression of genes belonging to expansin and Nrt gene families that were identified in the root of wheat grown under NDC were involved in increasing the root surface area and nitrogen transport. It can be regarded as the adaptation of wheat plants to increase nitrogen absorption (Fig. 9).

The expression of Pet, Psb, Nar, Nir gene family members were found to be down-regulated, which inhibited the rate of photosynthesis and nitrogen assimilation (Fig. 9). It hampered the biomass accumulation, culminating in reduced shoot height, leaf area, and root volume. Moreover, in the shoot of wheat plants grown under NDC, the down-regulated monobactam biosynthesis pathway with the highest enrichment score decreased antibacterial activity. Genetic engineering can be employed to increase the expression of down-regulated genes in four gene families (Pet, Psb, Nar, Nir) identified in our study. It can also increase the rate of photosynthesis and nitrogen metabolism to improve the matter accumulation and growth condition of crops grown under NDC.

## Conclusion

The wheat plants grown under the nitrogen-deficient conditions (NDC) showed reduced crop height, leaf area, root volume, photosynthetic rate, and crop weight and increased root length, root surface area, and root/shoot ratio as compared to control. 3949 (2414 down-regulated, 1535 up-regulated) differentially expressed genes (DEGs) were identified in the shoot, and 3911 (2675 down-regulated, 1536 up-regulated) DEGs were identified in the root of the wheat plants grown under NDC.

GO pathway and KEGG pathway enrichment analysis of these DEGs were also conducted. 24 expansin genes (such as treasCS5B02G528400) and 9 Nrt genes (such as TreasCS6A02G031100) were correlated to increased N absorption. Besides, 3 Pet genes (such as TreasCS7B02G226200) and 8 Psb genes (such as TreasCS3D02G523300) were correlated to the inhibition of the photosynthetic pathway; also, 3 Nar genes (such as TreasCS6A02G326200) and 1 Nir gene (TreasCS6D02G333900) were correlated to the inhibition of nitrogen metabolism pathway in wheat plants grown under NDC.

## Methods

### Experimental design

The nitrogen sensitive wheat cultivar, Shannong 29, was used in this study. The experiments were conducted in the Huang Huai Hai region, China. The seeds of Shannong 29 were procured from ShanDong Shofine Seed Technology Co., Ltd. Two nutrient solution with different nitrogen concentrations (NH_4_NO_3_), i.e., complete nutrient solution (N1) with 5 mmol L^− 1^ NH_4_NO_3_ and nutrient solution without nitrogen (N0) with 0 mmol L^− 1^ NH_4_NO_3_ (N0), were used in this study. Thus, wheat plants grown using nutrient solutions, N0 and N1, were referred to as N0 and N1 groups of plants, respectively. Hoagland solution formula was used to prepare a nutrient solution (N0: without nitrogen). The nutrient solution contained 2 mmol L^− 1^ CaCl_2_, 1.8 mmol L^− 1^ KCl, 0.2 mmol L^− 1^ KH_2_PO_4_, 0.5 mmol L^− 1^ MgSO_4_, 0.1 mmol L^− 1^ FeEDTA, 0.5 μmol L^− 1^ KI, 1 μmol L^− 1^ H_3_BO_3_, 1 μmol L^− 1^ MnSO_4_, 1 μmol L^− 1^ ZnSO_4_, 1 μmol L^− 1^ Na_2_MoO_4_, 0.1 μmol L^− 1^ CuSO_4_, 0.1 μmol L^− 1^ CoCl_2_. The pH was maintained at 6.8 ± 0.3. The wheat seeds were first sterilized with 75% alcohol for 30 s and later washed with sterilized distilled water three times. After sterilizing the winter wheat seeds, the seedlings were cultured to 1 leaf and 1 heart stage. Each experimental treatment contained 100 seedlings, and each experiment was repeated three times. The seedlings were transplanted to different nutrient solutions and fixed by sponges. The seedlings were cultured in an artificial incubator with 8 and 16 h of dark and light cycle, respectively, and 70% relative humidity.

### Experimental measurements

#### Morphological index

3 days after transplanting seedlings into nutrient solution, 10 plants in each treatment (repeated in three repetitions) were sampled for measuring leaf area, plant height, root length, root surface area and root volume. In addition, another 10 plants were sampled for determining fresh weight of root and shoot. The method for measuring root length, surface area and volume was followings: artificially rinse the roots, remove impurities and miscellaneous roots, absorb the surface water of the roots, spread the roots in the glass dish of the root scanner (0.24 × 0.32 m), and save the photos as 600 API pixels by the root scanner (HP Scanjet 8200; Hewlett-Packard, Palo Alto, CA, USA). The root analysis software (Delta-T Area Meter Type AMB2; Delta-T Devices Ltd., Cambridge, UK) was used for data analysis.

### Physiological index

3 days after transplanting seedlings into the nutrient solution, the net photosynthetic rate (Pn), stomatal conductance (Gs), and intercellular carbon dioxide concentration (Ci) of top leaves were measured using LI-6400 portable photosynthesizer (LI-COR, USA) with a red-blue light source and a light quantum density of 1400 μmol m^2^ s^-l^.

### Transcriptome sequencing

1 day after transplanting seedlings to the nutrient solution, 20 plants per experimental repetition were quickly sampled, followed by the separation of roots and shoots, and later these samples were put into liquid nitrogen for quick freezing. Total RNA was extracted using the mirVana miRNA isolation kit (Ambion), as per the manufacturer’s instruction. RNA integrity was evaluated using the Agilent 2100 Bioanalyzer (Agilent Technologies, Santa Clara, CA, USA). For subsequent analysis, only the samples with RNA Integrity Number (RIN) ≥ 7 were used. The libraries were constructed using TruSeq Stranded mRNA LTSample Prep Kit (Illumina, San Diego, CA, USA), according to the manufacturer’s instructions. These libraries were sequenced on the Illumina sequencing platform (HiSeqTM 2500 or Illumina HiSeq X Ten), and 125 bp/150 bp paired-end reads were generated.

### RT-qPCR based validation

The wheat shoots and roots were sampled at the same time for the transcriptome sequencing. These samples were immediately frozen on liquid nitrogen and stored at -80 °C. The 50–100 mg plant tissues from each sample were ground into powder in liquid nitrogen, and 500 μL buffer RLS was added to each of the powdered samples. The sample was mixed by centrifuge immediately. The RNA was extracted using an RNA kit (Kangwei, China). The PCR reaction mixture contained RNA 7 μL, Oligo (dT) 1 μL, 2*R-Mix 10 μL, E-mix 1 μL, gDNA remover 1 μL, Rnase-free water 0 μL. Primers were designed using NCBI’s premier blast. The real-time quantitative RT-PCR analysis was carried out by using a multi-channel fluorescent quantitative PCR instrument (CFX 384 Touch, America). TBtools was employed to construct the heatmaps [45].

### Data analysis

The differentially expressed genes (DEGs) were functionally categorized using Gene Ontology [46]. The genome and mRNA database used was ftp://ftp.ensemblgenomes.org/pub/plants/release-45/fasta/triticum_aestivum/dna/Triticum_aestivum.IWGSC.dna.toplevel.fa.gz. In GO functional enrichment analysis (http://geneontology.org/), all protein-coding genes/transcripts were used as background lists, and differential protein-coding genes/transcripts were used as candidate lists screened from background lists. A hypergeometric distribution test was employed to calculate the *p*-value. It represents the significance of the enriched GO functional category of differential protein-coding genes/transcripts. The p-value was tested using Benjamin & Hochberg’s multiple tests.

We used KEGG [47, 48] database (http://www.genome.jp/kegg/) to analyze the DEGs (combined with KEGG annotation results). Besides, the hypergeometric distribution test was used to calculate the significance of differential gene enrichment in each pathway. The calculated results will return a significant p-value of enrichment, and a small p-value indicated that the differential gene had been enriched in the pathway. KEGG pathway enrichment analysis was performed to unveil the enrichment of DEGs.

The experimental data were represented as the mean value from three replicates. Statistical calculations were performed using SPSS software version 19.0 (SPSS Inc., Chicago, USA). Experimental treatments were compared using one-way ANOVA and Duncan’s multiple range test (DMRT). *P* < 0.05 was considered statistically significant.

## Supplementary information

**Additional file 1.**

**Additional file 2.**

**Additional file 3.**

**Additional file 4.**

**Additional file 5.**

**Additional file 6.**

**Additional file 7.**

**Additional file 8.**

**Additional file 9.**

## Data Availability

The transcriptome data associated with this article were uploaded for supplementary data. Other datasets are available from the corresponding author on a reasonable request.

## Abbreviations

Psb: Photosystem II reaction center protein
Pet: Cytochrome b6/f complex subunit 4
Nar: Nitrate reductase
Nrt: High affinity nitrate transporter
Exp: Expansin
Pn: Net photosynthetic rate
Gs: Stomatal conductance
Ci: Intercellular carbon dioxide concentration
DEG: Differentially Expressed Genes
KEGG: Kyoto Encyclopedia of Genes and Genomes
GO: Gene Ontology
NDC: Nitrogen-Deficient Conditions
PCA: Principal Component Analysis
FPKM: Fragments Per Kilobase of transcript per Million fragments mapped
