# Supplementary figures and images for: Transcription strategies related to photosynthesis and nitrogen metabolism of wheat in response to nitrogen deficiency
Source: BMC Plant Biol. 2020 Oct 1;20:448. doi: 10.1186/s12870-020-02662-3 (PMC7528333; doi:10.1186/s12870-020-02662-3)

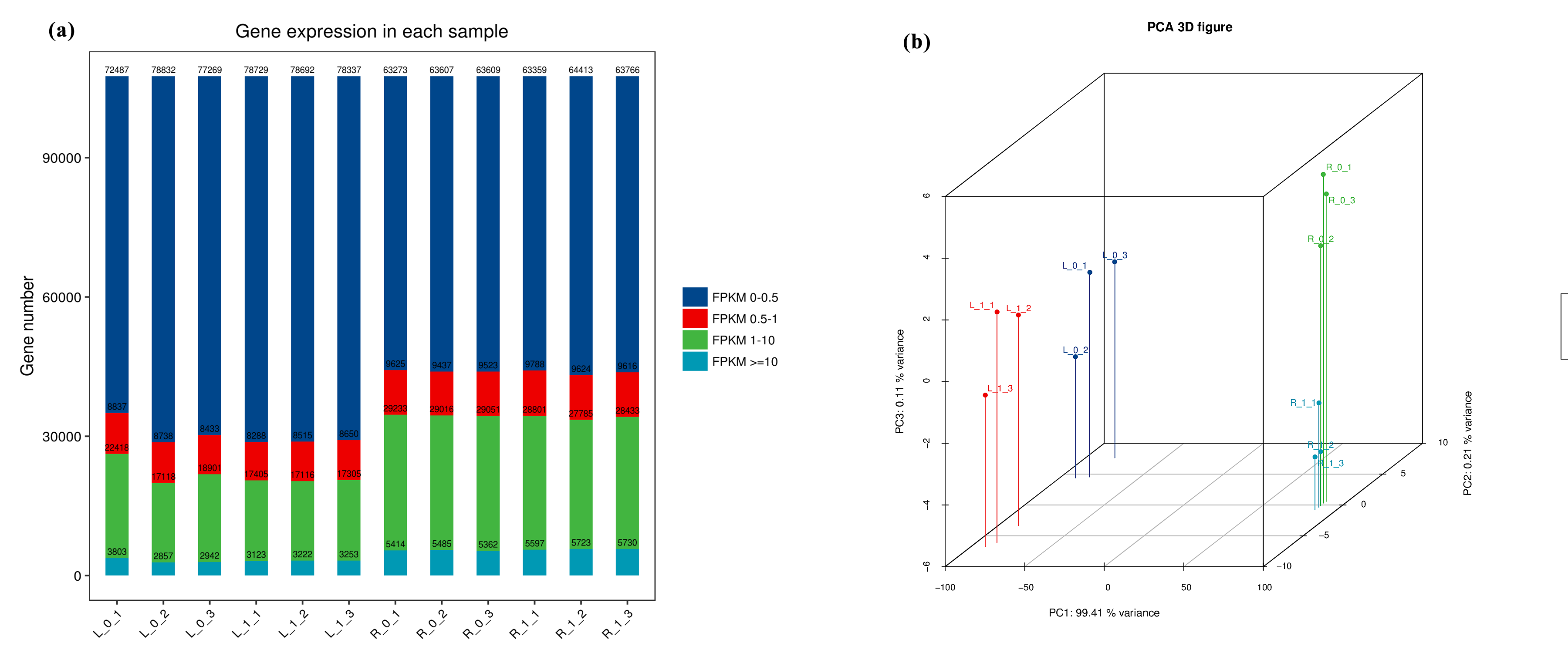

Supplement: Supplementary file 8 — Additional file 8. [file 12870_2020_2662_MOESM8_ESM.tif]
